# Bis(μ-diisopropyl­phosphanido-κ^2^ *P*:*P*)bis­[hydrido(triisopropyl­phosphane-κ*P*)platinum(II)]

**DOI:** 10.1107/S1600536812022829

**Published:** 2012-05-26

**Authors:** Nicole Arnold, Holger Braunschweig, Alexander Damme

**Affiliations:** a Institut für Anorganische Chemie, Universität Würzburg, Am Hubland, D-97074 Würzburg, Germany

## Abstract

In the centrosymmetric mol­ecular structure of the title compound [Pt_2_(C_6_H_14_P)_2_H_2_(C_9_H_21_P)_2_], each Pt^II^ atom is bound on one side to a phosphane ligand (P*i*Pr_3_) and a hydrido ligand. On the other side, it is bound to two phosphanide ligands (μ-P*i*Pr_2_), which engage a bridging position between the two Pt^II^ atoms, forming a distorted square-planar structure motif. The Pt⋯Pt distance is 3.6755 (2) Å. A comparable mol­ecular structure was observed for bis­(μ-di-*tert*-butyl­phosphanido)bis­[hydrido(triethyl­phosphane)platinum(II)] [Itazaki *et al.* (2004 ▶). *Organometallics*, **23**, 1610–1621].

## Related literature

For the syntheses of similar phosphido-bridged complexes of platinum(II) with phosphine ligands, see: Itazaki *et al.* (2004) ▶ or with other ligands such as carbonyl, see: Albinati *et al.* (2008 ▶). For Pt—H bond lengths in related structures, see: Chiang *et al.* (1984 ▶); Knobler *et al.* (1983 ▶).
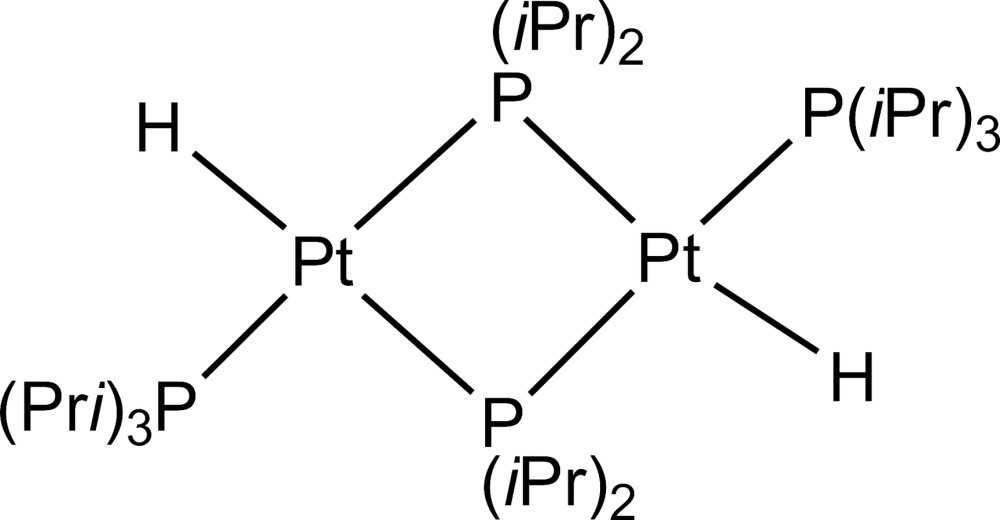


## Experimental

### 

#### Crystal data

[Pt_2_(C_6_H_14_P)_2_H_2_(C_9_H_21_P)_2_]
*M* *_r_* = 946.94
Monoclinic, 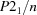

*a* = 8.8301 (3) Å
*b* = 14.8153 (5) Å
*c* = 14.1688 (5) Å
β = 90.097 (2)°
*V* = 1853.57 (11) Å^3^
*Z* = 2
Mo *K*α radiation
μ = 7.73 mm^−1^
*T* = 100 K
0.53 × 0.13 × 0.11 mm

#### Data collection

Bruker X8 APEXII diffractometer
Absorption correction: multi-scan (*SADABS*; Bruker, 2008 ▶) *T*_min_ = 0.360, *T*_max_ = 0.745
38282 measured reflections
3943 independent reflections
3478 reflections with *I* > 2σ(*I*)
*R*_int_ = 0.051

#### Refinement

*R*[*F* ^2^ > 2σ(*F* ^2^)] = 0.018
*wR*(*F* ^2^) = 0.038
*S* = 1.03
3943 reflections
177 parameters
H atoms treated by a mixture of independent and constrained refinement
Δρ_max_ = 0.72 e Å^−3^
Δρ_min_ = −0.66 e Å^−3^

### 

Data collection: *APEX2* (Bruker, 2010 ▶); cell refinement: *SAINT-Plus* (Bruker, 2010 ▶); data reduction: *SAINT-Plus*; program(s) used to solve structure: *SHELXS97* (Sheldrick, 2008 ▶); program(s) used to refine structure: *SHELXL97* (Sheldrick, 2008 ▶); molecular graphics: *XP* in *SHELXTL* (Sheldrick, 2008 ▶); software used to prepare material for publication: *SHELXL97*.

## Supplementary Material

Crystal structure: contains datablock(s) I, global. DOI: 10.1107/S1600536812022829/hp2036sup1.cif

Structure factors: contains datablock(s) I. DOI: 10.1107/S1600536812022829/hp2036Isup2.hkl

Additional supplementary materials:  crystallographic information; 3D view; checkCIF report

## Figures and Tables

**Fig. 1.**
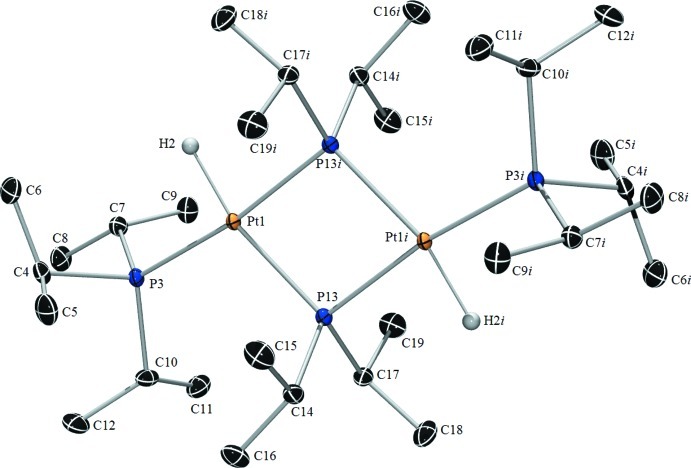
The molecular structure of the title compound showing the atom numbering scheme and displacement ellipsoides for the non-H atoms at the 50% probability level. Hydrogen atoms are omitted for clarity.

## supplementary crystallographic information

### Comment

Bis[µ-di(*iso*propyl)phosphino]-di(hydrido)-bis[tri(*iso*propyl)phosphine]-di(platinum), bridged by the µ-P*i*Pr_2_ ligands, displays a slightly distorted square-planar geometry. The two platinum centers show a Pt(1)–Pt(1*^i^*) distance of 3.6755 (2) Å. The Pt–Pt distance is comparable to that in bis[µ-di(*tert*-butyl)phosphino]-di(hydrido)-bis[tri(ethyl)phosphine]-di(platinum) [Pt_2_H_2_(µ-P*^t^*Bu_2_)_2_(PEt_3_)_2_] (3.646 Å).

The bond angles P(13)–Pt(1)–P(13^i^) [77.47 (3)°] and Pt(1)–P(13)–Pt(1^i^) [102.53 (3)°] are slightly out of range of the structural parameters of the complexes without Pt–Pt bonding from Itazaki *et al.* (2004) [P–Pt–P 74.6–77.2° and Pt–P–Pt 102.8–105.4°]. This could be due to the less sterical hindrance of the *iso-*propyl groups by contrast with the *tert*-butyl groups in the reference substance [Pt_2_H_2_(µ-P*^t^*Bu_2_)_2_(PEt_3_)_2_].

Chiang *et al.* (1984) reported the bond length of a terminal Pt–H bond determined by neutron diffraction method. They found for the Pt–H bond on a five coordinate platinum centre a bond length of 1.610 (2) Å in the compound [Pt_2_H_3_(Ph_2_PCH_2_CH_2_PPh_2_)_2_]^+^[BPh_4_]^-^. In the title compound [Pt_2_H_2_(µ-P*i*Pr_2_)_2_(P*i*Pr_3_)_2_] [1.57 (3) Å] the bonding disctance of Pt–H is 2.5% shorter than in the neutron experiment of Chiang *et al.*, due to the smaller coordination number of four in the former species.

The group of Knobler *et al.* (1983) also determined the Pt–H bond length by X-Ray diffraction in [Pt_2_H_3_(Ph_2_PCH_2_CH_2_PPh_2_)_2_]^+^[BPh_4_]^-^ to be 1.527 Å, however without further refinement.

The bonding dictances Pt–P in *trans-*position to the hydrido ligand are with 2.3773 (7) Å longer than the bonding distances in *trans-*position to the phosphine ligand 2.3343 (7) Å.

### Experimental

Bis(tri-*iso*-propylphosphine)platinum (50.0 mg, 0.09 mmol) dissolved in 1 ml benzene was added to a solution of dichloro(2,3,5,6-tetramethylphenyl)borane (29.5 mg, 0.09 mmol) in 1 ml benzene. The solvent was removed under reduced pressure and the obtained dark brown residue was disolved in hexanes. The title compound was obtained as a off-white solid. Colourless crystals suitable for X-ray analysis were grown from a hexanes solution at 238 K.

### Refinement

The H atoms were placed at idealized positions and treatet as riding atoms: C–H = 0.98 Å (CH_3_), 1.00 Å (aliphatic H-atoms). *U*_iso_(H) values were fixed at 1.5 times (for primary H atoms) and 1.2 times (tertiary H atoms) *U*_eq_ of the attached C atoms.

### Crystal data

**Table:**

[Pt_2_(C_6_H_14_P)_2_H_2_(C_9_H_21_P)_2_]	*F*(000) = 936
*M**_r_* = 946.94	*D*_x_ = 1.697 Mg m^−^^3^
Monoclinic, *P*2_1_/*n*	Mo *K*α radiation, λ = 0.71073 Å
Hall symbol: -P 2yn	Cell parameters from 8162 reflections
*a* = 8.8301 (3) Å	θ = 2.7–26.7°
*b* = 14.8153 (5) Å	µ = 7.73 mm^−^^1^
*c* = 14.1688 (5) Å	*T* = 100 K
β = 90.097 (2)°	Needle, colourless
*V* = 1853.57 (11) Å^3^	0.53 × 0.13 × 0.11 mm
*Z* = 2	

### Data collection

**Table:**

Bruker X8 APEXII diffractometer	3943 independent reflections
Radiation source: rotating anode	3478 reflections with *I* > 2σ(*I*)
Multi-layer mirror monochromator	*R*_int_ = 0.051
Detector resolution: 8.333 pixels mm^-1^	θ_max_ = 26.8°, θ_min_ = 2.0°
φ and ω scans	*h* = −11→11
Absorption correction: multi-scan (*SADABS*; Bruker, 2008)	*k* = −18→18
*T*_min_ = 0.360, *T*_max_ = 0.745	*l* = −17→17
38282 measured reflections	

### Refinement

**Table:**

Refinement on *F*^2^	Primary atom site location: structure-invariant direct methods
Least-squares matrix: full	Secondary atom site location: difference Fourier map
*R*[*F*^2^ > 2σ(*F*^2^)] = 0.018	Hydrogen site location: inferred from neighbouring sites
*wR*(*F*^2^) = 0.038	H atoms treated by a mixture of independent and constrained refinement
*S* = 1.03	*w* = 1/[σ^2^(*F*_o_^2^) + (0.0141*P*)^2^ + 0.9947*P*] where *P* = (*F*_o_^2^ + 2*F*_c_^2^)/3
3943 reflections	(Δ/σ)_max_ = 0.009
177 parameters	Δρ_max_ = 0.72 e Å^−^^3^
0 restraints	Δρ_min_ = −0.66 e Å^−^^3^

### Special details

**Table:**

Experimental. The crystal was immersed in a film of perfluoropolyether oil, mounted on a polyimide microloop (MicroMounts of MiTeGen) and transferred to stream of cold nitrogen (Oxford Cryostream 700).
Geometry. All e.s.d.'s (except the e.s.d. in the dihedral angle between two l.s. planes) are estimated using the full covariance matrix. The cell e.s.d.'s are taken into account individually in the estimation of e.s.d.'s in distances, angles and torsion angles; correlations between e.s.d.'s in cell parameters are only used when they are defined by crystal symmetry. An approximate (isotropic) treatment of cell e.s.d.'s is used for estimating e.s.d.'s involving l.s. planes.
Refinement. Refinement of *F*^2^ against ALL reflections. The weighted *R*-factor *wR* and goodness of fit *S* are based on *F*^2^, conventional *R*-factors *R* are based on *F*, with *F* set to zero for negative *F*^2^. The threshold expression of *F*^2^ > σ(*F*^2^) is used only for calculating *R*-factors(gt) *etc*. and is not relevant to the choice of reflections for refinement. *R*-factors based on *F*^2^ are statistically about twice as large as those based on *F*, and *R*- factors based on ALL data will be even larger.

### Fractional atomic coordinates and isotropic or equivalent isotropic displacement parameters (Å^2^)

**Table:**

	*x*	*y*	*z*	*U*_iso_*/*U*_eq_	
Pt1	0.068246 (11)	1.032840 (7)	0.882448 (6)	0.00916 (4)	
P3	0.15611 (8)	0.99092 (5)	0.73662 (5)	0.01104 (15)	
C4	0.0188 (3)	1.01911 (19)	0.64139 (19)	0.0155 (6)	
H4	0.0570	0.9925	0.5811	0.019*	
C5	−0.1344 (3)	0.9764 (2)	0.6632 (2)	0.0230 (7)	
H5A	−0.1717	0.9993	0.7238	0.034*	
H5B	−0.1233	0.9107	0.6667	0.034*	
H5C	−0.2067	0.9918	0.6132	0.034*	
C6	0.0005 (3)	1.1205 (2)	0.6271 (2)	0.0218 (7)	
H6A	−0.0818	1.1318	0.5822	0.033*	
H6B	0.0951	1.1457	0.6023	0.033*	
H6C	−0.0234	1.1491	0.6876	0.033*	
C7	0.3257 (3)	1.05748 (19)	0.70284 (18)	0.0141 (6)	
H7	0.2888	1.1209	0.6953	0.017*	
C8	0.3981 (3)	1.0330 (2)	0.60752 (19)	0.0195 (7)	
H8A	0.4768	1.0773	0.5921	0.029*	
H8B	0.3202	1.0334	0.5581	0.029*	
H8C	0.4434	0.9727	0.6116	0.029*	
C9	0.4449 (3)	1.0616 (2)	0.7817 (2)	0.0199 (7)	
H9A	0.4974	1.0034	0.7859	0.030*	
H9B	0.3951	1.0747	0.8419	0.030*	
H9C	0.5184	1.1093	0.7675	0.030*	
C10	0.1983 (3)	0.86919 (18)	0.71777 (19)	0.0156 (6)	
H10	0.1209	0.8361	0.7558	0.019*	
C11	0.3510 (3)	0.8423 (2)	0.7605 (2)	0.0212 (7)	
H11A	0.3587	0.7763	0.7627	0.032*	
H11B	0.3591	0.8667	0.8246	0.032*	
H11C	0.4331	0.8666	0.7215	0.032*	
C12	0.1825 (4)	0.8318 (2)	0.6177 (2)	0.0235 (7)	
H12A	0.2552	0.8620	0.5761	0.035*	
H12B	0.0794	0.8427	0.5946	0.035*	
H12C	0.2028	0.7668	0.6181	0.035*	
P13	0.00800 (7)	0.90365 (5)	0.97455 (5)	0.01004 (14)	
C14	−0.1496 (3)	0.82851 (18)	0.93640 (18)	0.0141 (6)	
H14	−0.1686	0.7858	0.9897	0.017*	
C15	−0.2950 (3)	0.8827 (2)	0.9230 (2)	0.0220 (7)	
H15A	−0.2809	0.9267	0.8721	0.033*	
H15B	−0.3194	0.9145	0.9817	0.033*	
H15C	−0.3782	0.8418	0.9066	0.033*	
C16	−0.1193 (3)	0.7706 (2)	0.8494 (2)	0.0197 (7)	
H16A	−0.2015	0.7268	0.8415	0.030*	
H16B	−0.0231	0.7385	0.8573	0.030*	
H16C	−0.1139	0.8094	0.7934	0.030*	
C17	0.1654 (3)	0.82156 (18)	0.99243 (18)	0.0129 (6)	
H17	0.1924	0.7960	0.9293	0.015*	
C18	0.1266 (3)	0.7430 (2)	1.0573 (2)	0.0222 (7)	
H18A	0.2147	0.7032	1.0633	0.033*	
H18B	0.0414	0.7090	1.0307	0.033*	
H18C	0.0987	0.7662	1.1197	0.033*	
C19	0.3042 (3)	0.8713 (2)	1.0303 (2)	0.0216 (7)	
H19A	0.2810	0.8971	1.0923	0.032*	
H19B	0.3318	0.9199	0.9866	0.032*	
H19C	0.3890	0.8290	1.0362	0.032*	
H2	0.099 (3)	1.131 (2)	0.845 (2)	0.040 (9)*	

### Atomic displacement parameters (Å^2^)

**Table:**

	*U*^11^	*U*^22^	*U*^33^	*U*^12^	*U*^13^	*U*^23^
Pt1	0.01140 (6)	0.00952 (6)	0.00656 (6)	0.00047 (4)	0.00126 (4)	−0.00011 (4)
P3	0.0133 (4)	0.0122 (4)	0.0076 (3)	0.0013 (3)	0.0004 (3)	−0.0003 (3)
C4	0.0205 (16)	0.0180 (16)	0.0081 (13)	0.0010 (12)	−0.0003 (11)	−0.0021 (11)
C5	0.0195 (17)	0.0312 (19)	0.0182 (15)	−0.0033 (14)	−0.0068 (13)	−0.0003 (13)
C6	0.0242 (17)	0.0225 (18)	0.0187 (15)	0.0058 (14)	−0.0075 (12)	0.0020 (13)
C7	0.0161 (15)	0.0142 (15)	0.0118 (14)	−0.0016 (12)	0.0010 (11)	0.0002 (11)
C8	0.0206 (16)	0.0240 (18)	0.0140 (15)	−0.0027 (13)	0.0056 (12)	0.0024 (12)
C9	0.0155 (16)	0.0264 (17)	0.0179 (15)	−0.0036 (13)	−0.0011 (12)	0.0028 (13)
C10	0.0203 (16)	0.0105 (14)	0.0160 (14)	0.0000 (12)	0.0059 (12)	−0.0014 (12)
C11	0.0253 (17)	0.0175 (17)	0.0208 (16)	0.0049 (13)	0.0050 (13)	0.0028 (13)
C12	0.0338 (19)	0.0163 (16)	0.0204 (16)	0.0022 (14)	0.0037 (13)	−0.0076 (13)
P13	0.0118 (4)	0.0099 (3)	0.0085 (3)	0.0007 (3)	0.0010 (3)	−0.0005 (3)
C14	0.0178 (15)	0.0125 (15)	0.0121 (14)	−0.0047 (12)	0.0017 (11)	−0.0003 (11)
C15	0.0154 (16)	0.0255 (18)	0.0250 (16)	−0.0012 (13)	−0.0018 (12)	−0.0051 (14)
C16	0.0186 (16)	0.0195 (17)	0.0209 (15)	−0.0076 (13)	0.0024 (12)	−0.0062 (13)
C17	0.0150 (15)	0.0111 (15)	0.0125 (13)	0.0039 (11)	0.0023 (11)	−0.0006 (11)
C18	0.0283 (18)	0.0182 (17)	0.0201 (16)	0.0104 (14)	0.0030 (13)	0.0050 (13)
C19	0.0155 (16)	0.0229 (17)	0.0265 (17)	0.0036 (13)	−0.0028 (12)	−0.0021 (14)

### Geometric parameters (Å, º)

**Table:**

Pt1—P3	2.2940 (7)	C11—H11A	0.9800
Pt1—P13^i^	2.3343 (7)	C11—H11B	0.9800
Pt1—P13	2.3773 (7)	C11—H11C	0.9800
Pt1—H2	1.57 (3)	C12—H12A	0.9800
P3—C7	1.857 (3)	C12—H12B	0.9800
P3—C4	1.860 (3)	C12—H12C	0.9800
P3—C10	1.861 (3)	P13—C14	1.862 (3)
C4—C6	1.524 (4)	P13—C17	1.864 (3)
C4—C5	1.526 (4)	P13—Pt1^i^	2.3343 (7)
C4—H4	1.0000	C14—C16	1.526 (4)
C5—H5A	0.9800	C14—C15	1.526 (4)
C5—H5B	0.9800	C14—H14	1.0000
C5—H5C	0.9800	C15—H15A	0.9800
C6—H6A	0.9800	C15—H15B	0.9800
C6—H6B	0.9800	C15—H15C	0.9800
C6—H6C	0.9800	C16—H16A	0.9800
C7—C9	1.534 (4)	C16—H16B	0.9800
C7—C8	1.538 (4)	C16—H16C	0.9800
C7—H7	1.0000	C17—C18	1.523 (4)
C8—H8A	0.9800	C17—C19	1.527 (4)
C8—H8B	0.9800	C17—H17	1.0000
C8—H8C	0.9800	C18—H18A	0.9800
C9—H9A	0.9800	C18—H18B	0.9800
C9—H9B	0.9800	C18—H18C	0.9800
C9—H9C	0.9800	C19—H19A	0.9800
C10—C12	1.529 (4)	C19—H19B	0.9800
C10—C11	1.530 (4)	C19—H19C	0.9800
C10—H10	1.0000		
			
P3—Pt1—P13^i^	171.67 (3)	C10—C11—H11A	109.5
P3—Pt1—P13	110.66 (2)	C10—C11—H11B	109.5
P13^i^—Pt1—P13	77.47 (3)	H11A—C11—H11B	109.5
P3—Pt1—H2	83.5 (12)	C10—C11—H11C	109.5
P13^i^—Pt1—H2	88.4 (12)	H11A—C11—H11C	109.5
P13—Pt1—H2	165.8 (12)	H11B—C11—H11C	109.5
C7—P3—C4	102.65 (13)	C10—C12—H12A	109.5
C7—P3—C10	108.41 (13)	C10—C12—H12B	109.5
C4—P3—C10	104.12 (13)	H12A—C12—H12B	109.5
C7—P3—Pt1	111.26 (9)	C10—C12—H12C	109.5
C4—P3—Pt1	111.81 (9)	H12A—C12—H12C	109.5
C10—P3—Pt1	117.37 (9)	H12B—C12—H12C	109.5
C6—C4—C5	110.0 (2)	C14—P13—C17	101.90 (12)
C6—C4—P3	112.7 (2)	C14—P13—Pt1^i^	106.05 (9)
C5—C4—P3	109.69 (19)	C17—P13—Pt1^i^	111.17 (9)
C6—C4—H4	108.1	C14—P13—Pt1	119.34 (9)
C5—C4—H4	108.1	C17—P13—Pt1	115.63 (9)
P3—C4—H4	108.1	Pt1^i^—P13—Pt1	102.53 (3)
C4—C5—H5A	109.5	C16—C14—C15	110.1 (2)
C4—C5—H5B	109.5	C16—C14—P13	116.01 (19)
H5A—C5—H5B	109.5	C15—C14—P13	110.48 (19)
C4—C5—H5C	109.5	C16—C14—H14	106.6
H5A—C5—H5C	109.5	C15—C14—H14	106.6
H5B—C5—H5C	109.5	P13—C14—H14	106.6
C4—C6—H6A	109.5	C14—C15—H15A	109.5
C4—C6—H6B	109.5	C14—C15—H15B	109.5
H6A—C6—H6B	109.5	H15A—C15—H15B	109.5
C4—C6—H6C	109.5	C14—C15—H15C	109.5
H6A—C6—H6C	109.5	H15A—C15—H15C	109.5
H6B—C6—H6C	109.5	H15B—C15—H15C	109.5
C9—C7—C8	111.3 (2)	C14—C16—H16A	109.5
C9—C7—P3	112.72 (19)	C14—C16—H16B	109.5
C8—C7—P3	115.95 (19)	H16A—C16—H16B	109.5
C9—C7—H7	105.3	C14—C16—H16C	109.5
C8—C7—H7	105.3	H16A—C16—H16C	109.5
P3—C7—H7	105.3	H16B—C16—H16C	109.5
C7—C8—H8A	109.5	C18—C17—C19	109.8 (2)
C7—C8—H8B	109.5	C18—C17—P13	114.37 (19)
H8A—C8—H8B	109.5	C19—C17—P13	109.31 (19)
C7—C8—H8C	109.5	C18—C17—H17	107.7
H8A—C8—H8C	109.5	C19—C17—H17	107.7
H8B—C8—H8C	109.5	P13—C17—H17	107.7
C7—C9—H9A	109.5	C17—C18—H18A	109.5
C7—C9—H9B	109.5	C17—C18—H18B	109.5
H9A—C9—H9B	109.5	H18A—C18—H18B	109.5
C7—C9—H9C	109.5	C17—C18—H18C	109.5
H9A—C9—H9C	109.5	H18A—C18—H18C	109.5
H9B—C9—H9C	109.5	H18B—C18—H18C	109.5
C12—C10—C11	110.6 (2)	C17—C19—H19A	109.5
C12—C10—P3	117.8 (2)	C17—C19—H19B	109.5
C11—C10—P3	111.9 (2)	H19A—C19—H19B	109.5
C12—C10—H10	105.1	C17—C19—H19C	109.5
C11—C10—H10	105.1	H19A—C19—H19C	109.5
P3—C10—H10	105.1	H19B—C19—H19C	109.5
			
P13^i^—Pt1—P3—C7	31.4 (2)	C7—P3—C10—C11	48.1 (2)
P13—Pt1—P3—C7	−135.55 (10)	C4—P3—C10—C11	156.87 (19)
P13^i^—Pt1—P3—C4	−82.7 (2)	Pt1—P3—C10—C11	−79.0 (2)
P13—Pt1—P3—C4	110.32 (10)	P3—Pt1—P13—C14	−65.19 (10)
P13^i^—Pt1—P3—C10	157.11 (18)	P13^i^—Pt1—P13—C14	116.73 (10)
P13—Pt1—P3—C10	−9.88 (11)	P3—Pt1—P13—C17	56.96 (10)
C7—P3—C4—C6	−51.8 (2)	P13^i^—Pt1—P13—C17	−121.13 (10)
C10—P3—C4—C6	−164.7 (2)	P3—Pt1—P13—Pt1^i^	178.09 (2)
Pt1—P3—C4—C6	67.6 (2)	P13^i^—Pt1—P13—Pt1^i^	0.0
C7—P3—C4—C5	−174.7 (2)	C17—P13—C14—C16	−57.0 (2)
C10—P3—C4—C5	72.3 (2)	Pt1^i^—P13—C14—C16	−173.41 (19)
Pt1—P3—C4—C5	−55.3 (2)	Pt1—P13—C14—C16	71.7 (2)
C4—P3—C7—C9	167.9 (2)	C17—P13—C14—C15	176.81 (19)
C10—P3—C7—C9	−82.4 (2)	Pt1^i^—P13—C14—C15	60.41 (19)
Pt1—P3—C7—C9	48.1 (2)	Pt1—P13—C14—C15	−54.5 (2)
C4—P3—C7—C8	−62.2 (2)	C14—P13—C17—C18	−53.1 (2)
C10—P3—C7—C8	47.5 (2)	Pt1^i^—P13—C17—C18	59.6 (2)
Pt1—P3—C7—C8	178.02 (18)	Pt1—P13—C17—C18	175.91 (17)
C7—P3—C10—C12	−81.7 (2)	C14—P13—C17—C19	−176.60 (19)
C4—P3—C10—C12	27.1 (3)	Pt1^i^—P13—C17—C19	−63.98 (19)
Pt1—P3—C10—C12	151.27 (19)	Pt1—P13—C17—C19	52.4 (2)

Symmetry code: (i) −*x*, −*y*+2, −*z*+2.

## References

[bb1] Albinati, A., Leoni, P., Marchetti, F., Marchetti, L., Pasquali, M. & Rizzato, S. (2008). *Eur. J. Inorg. Chem.* pp. 4092–4100.

[bb2] Bruker (2008). *SADABS* Bruker AXS Inc., Madison, Wisconsin, USA.

[bb3] Bruker (2010). *APEX2* and *SAINT-Plus* Bruker AXS Inc., Madison, Wisconsin, USA.

[bb4] Chiang, M. Y., Bau, R., Minghetti, G., Bandini, A. L., Banditelli, G. & Koetzle, T. F. (1984). *Inorg. Chem.* **23**, 122–124.

[bb5] Itazaki, M., Nishihara, Y. & Osakada, K. (2004). *Organometallics*, **23**, 1610–1621.

[bb6] Knobler, C. B., Kaesz, H. D., Minghetti, G., Bandini, A. L. & Banditelli, F. B. (1983). *Inorg. Chem.* **22**, 2324–2331.

[bb7] Sheldrick, G. M. (2008). *Acta Cryst.* A**64**, 112–122. 10.1107/S0108767307043930 18156677

